# Management of pneumothorax in hemodynamically stable preterm infants using high frequency oscillatory ventilation: report of five cases

**DOI:** 10.1186/s13052-017-0436-y

**Published:** 2017-12-22

**Authors:** Claudia Aurilia, Cinzia Ricci, Milena Tana, Chiara Tirone, Alessandra Lio, Alessandro Gambacorta, Angela Paladini, Giovanni Vento

**Affiliations:** Division of Neonatology, Department for the protection of women’s health and the nascent life, child and adolescent, Fondazione Policlinico Universitario A. Gemelli-Università Cattolica del Sacro Cuore, Largo A. Gemelli 8, 00168 Rome, Italy

**Keywords:** Pneumothorax, Preterm infants, HFOV, Chest drainage

## Abstract

**Background:**

Despite an increased use of non-invasive ventilatory strategies and gentle ventilation, pneumothorax remains a common complication in preterm infants. The ventilator management of infants with air leaks remains challenging in terms of both prevention and treatment. Recently the safety and efficacy of expectant management avoiding chest tube drainage to treat large air leak in preterm infants hemodynamically stable has been reported.

**Case presentation:**

In the present study, we report five cases of preterm infants with birth weight ≤ 1250 g affected by respiratory distress syndrome and treated with nasal continuous positive airway pressure as first intention. They were intubated for worsening of respiratory distress with increasing oxygen requirement and concomitant increase of respiratory rate and P_CO2_ values due to occurrence of pneumothorax, and they were successfully treated using high-frequency oscillatory ventilation without chest tube insertion.

**Conclusion:**

In our experience high-frequency oscillatory ventilation provided a conservative management of a significant pneumothorax in preterm newborns hemodynamically stable and requiring mechanical ventilation. This approach allowed us to avoid the increasing of air leak and the insertion of chest tube drainage and all the subsequent associated risks.

## Background

Pneumothorax is a relatively common complication in newborns infants (6–10% in very low birth weight preterm infants and around 1% in term infants [1]), especially when receiving invasive mechanical ventilation, but also during non-invasive ventilatory support such as continuous positive airways pressure (CPAP) [2]. A 40% increase of FiO_2_ during the first 24 h of CPAP has been suggested as a useful marker to identify the infants at high risk of developing a pneumothorax [3]. Recently successful management strategies have been reported of symptomatic pneumothorax diagnosed in preterm infants receiving assisted ventilation, which avoided chest tube drainage and resulted in reduction of important side effects [4, 5]. In this case series, we reported our experience of using high-frequency oscillatory ventilation (HFOV) as a first-line treatment of pneumothorax in preterm infants with respiratory distress syndrome.

## Cases presentation

We report the experience of five neonates (4 males and 1 female), with mean ± SD birth weight of 1211 ± 40 g and mean ± SD gestational age of 30 ± 2 weeks. All infants were delivered by caesarean section due to preeclampsia (one case), gestational hepatosis and preeclampsia (one case), preterm labor (one case), growth restriction (one case) and ascites of the second twin (one case); three of them were twin-birth. Premature rupture of membranes did not occur in any of the cases. All the mothers received at least one dose of betamethasone. Soon after birth, all the infants developed respiratory distress (Silverman score 3–4) and were placed on a ventilator nasal CPAP system with a pressure level of 4–6 cmH_2_O and a FiO_2_ of 0.25–0.30 for a diagnosis of respiratory distress syndrome based also on the typical chest X-ray. CPAP failure criteria (FiO_2_ ≥ 0.40) was reached in two cases (patient 3 and 5), and surfactant was administered (200 mg/kg of poractant alfa, Chiesi Farmaceutici, Italy) with the INtubation-SURfactant-Extubation (INSURE) approach respectively at 9 h and 6 h of life.

**Fig. 1:**
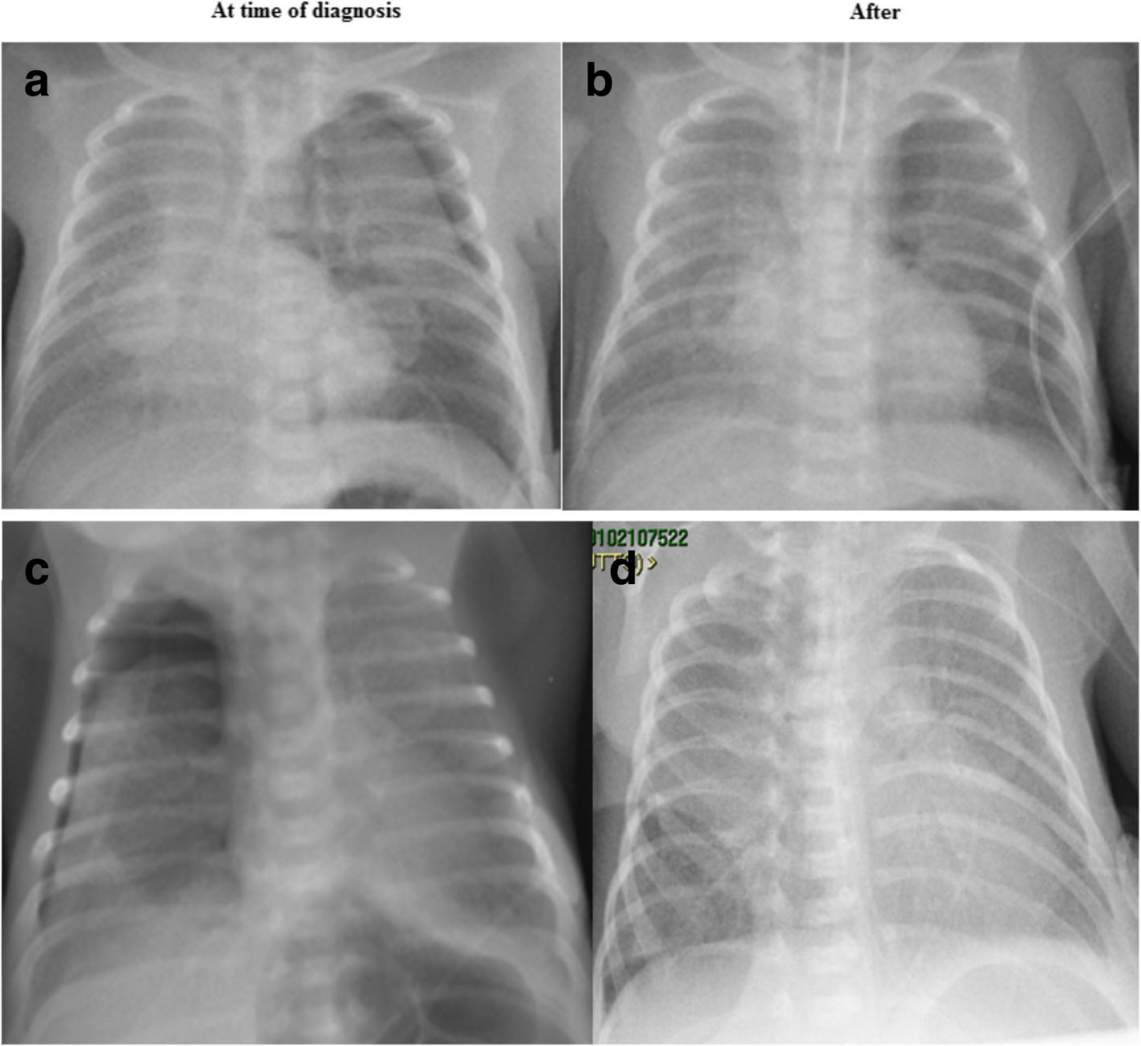
Chest X Rays of patient 1 and patient 4. Legend: patient 1: **a**: left side pneumothorax. **b**: resolution of pneumothorax 48 h later; patient 4: **c**: right side pneumothorax. **d**: resolution of pneumothorax 48 h later

On median [range] postnatal day 3 [2–4] respiratory distress (quantified by means of Silverman score) worsened, with a concomitant increase of respiratory rate, of capillary P_CO2_ values and of FiO_2_ to obtain SpO_2_ values 90–95% (Table 1). Chest X-rays showed the presence of pneumothorax on the right side in two patients and on the left side in the other three ones. Heart rate and blood pressure remained within normal range for the gestational age. All 5 neonates were intubated and mechanically ventilated using HFOV delivered by a BabyLog 8000 plus ventilator (Dräger, Lubeck, Germany) in the first 4 cases and by a BabyLog VN500 (Dräger, Lubeck, Germany) in the 5th case (Table 1). Ventilation was started with a median [range] mean airways pressure (MAP) of 8 [8-9] cmH_2_O, amplitude of 100% in the first 4 cases and ΔP of 15 cm H_2_O in the last case ventilated with Baby-log VN500, frequency of 10 Hz. The oscillation amplitude with the BabyLog 8000 plus is adjustable as a percentage from 0 to 100%, where 100% means the highest possible amplitude under the given circumstances of MAP and frequency settings as well as the characteristics of the respiratory system. In the first 4 cases receiving HFOV by BabyLog 8000 plus, amplitude was gradually increased up to 100% until the infant’s chest was seen to be “visibly vibrating”, providing the MAP as low as possible for the presence of air leak. All the newborns received Remifentanil by continuous intravenous infusion at a dose of 0.075 μg/kg/min to provide analgesia and sedation during HFOV [6]. Despite the start of mechanical ventilation, the FiO_2_ requirement remained quite high (0.50–0.60) in two cases (patients 2 and 3) and these neonates received 1 dose of surfactant (Table 1). In two patients, a diagnosis of pneumonia was made afterwards, based on the results of bronchoalveolar lavage fluid culture positive for *Candida spp.* (patient3) and for *Group B Streptococcus* (patient 4) (Table 1). In all the five cases, the infants’ oxygen requirement and ventilatory support gradually decreased during the subsequent days. Chest X-rays showed a progressive and complete resolution of the pneumothorax within 48–96 h (Fig. 1). All the infants were successfully extubated with a median [range] MAP of 6 [5.5–6] cmH_2_O, amplitude of 100 [25–100] % in the first 4 cases and ΔP of 15 cm H_2_O in the last case, frequency of 8 [7-10] Hz and FiO_2_ of 0.23 [0.21–0.25]. All infants survived with normal head ultrasounds and without bronchopulmonary dysplasia, defined as oxygen requirements or need of ventilatory support at 36 weeks of post menstrual age.

**Table 1:** Patients characteristics, pneumothorax details, respiratory status prior to intubation and starting ventilatory parameters

C	Patient 1	Patient 2	Patient 3	Patient 4	Patient 5
Gestational Age (weeks)	31^+6^	30^+2^	27^+5^	33^+2^	28^+5^
Sex	Male	Male	Female	Male	Male
Birth Weight (g)	1240	1215	1205	1250	1145
Delivery	Caesarean section	Caesarean section	Caesarean section	Caesarean section	Caesarean section
Respiratory Diagnosis	Respiratory Distress Syndrome	Respiratory Distress Syndrome	Respiratory Distress Syndrome; *Candida spp.* pneumonia	Respiratory Distress Syndrome; *Group B Streptococcus* pneumonia	Respiratory Distress Syndrome
Time of Surfactant	No	At 36 h of life, after intubation	INSURE at 9 h of life; 2nd dose at 50 h of life, after intubation	No	INSURE at 6 h of life
Pneumothorax diagnosis (day of life)	3	2	3	4	3
Site of the pneumothorax	Left	Left	Right	Right	Left
Pneumothorax resolution (day of life)	5	4	7	6	6
Extubation (day of life)	5	4	8	6	6
CPAP level (cmH_2_O)	4	4	6	4	4
Maximum FiO_2_	0.50	0.70	0.60	0.50	0.50
Maximum Silverman score	6	6	5	7	4
Spontaneous respiratory rate (breaths per minute)	95	110	75	110	90
pH, capillary blood	7.22	7.22	7.29	7.27	7.29
P_CO2_ (mmHg), capillary blood	58	64	59	64	60
MAP (cmH_2_O)	8	9	8	8	8
Frequency (Hz)	10	10	10	10	10
FiO_2_	0.50	0.50	0.50	0.50	0.50
Amplitude, (% or cmH_2_O^a^)	100	100	100	100	15^a^

MAP: mean airways pressure; CPAP: continuous positive airway pressure;

INSURE: INtubation-SURfactant-Extubation

^a^HFOV delivered by BabyLog VN500

## Discussion and conclusion

Despite an increased use of non-invasive ventilator strategies and gentle ventilation, pneumothorax remains a common complication in preterm infants. The ventilator management of infants with air leaks remains challenging in terms of both prevention and treatment. Several reports have described the successful application of high frequency ventilation (HFV) in adults with airway disruption or bronchopleural fistulae and in newborns with persistent air leaks through pneumothorax [7, 8]. Gonzalez et al. showed a decreased gas flow through chest tube insertion in neonates affected by pneumothorax receiving high-frequency jet ventilation with respect to those who received conventional ventilation [7]. Various forms of HFV have been used to treat infants with pulmonary air leak and the superiority of this type of ventilation over conventional ventilation has been shown [9]. It is not clear how HFV improves the healing of air leaks, but a ventilator strategy incorporating short inspiratory time and high respiratory rates is often effective in decreasing the magnitude of the leak [7, 8, 10]. It is most likely that the absence of high-peak inspiratory pressures, the very short absolute inspiratory time and small tidal volume applied at higher frequencies may result in a rapid decrease of air leak, as showed by Ellsbury et al. in an animal model of pneumothorax [11]. For these reasons, in the presence of gross air leak (e.g. pneumothorax), strategy should prioritise low tidal volume ventilation, more easily obtained during HFOV (1.5–2.5 ml/kg) respect to conventional mechanical ventilation (4–6 ml/kg). Management of MAP is also a critical tool and aggressive lung volume recruitment has to be avoided. MAP should be reduced where possible then maintained at a pressure sufficient to stent small airways open to avoid progression of the air leak and to guarantee sufficient oxygenation without high FiO_2_ requirements [12].

Recently Kitsommart et al. [4] reported 4 cases of preterm infants who developed large pneumothoraces, two of whom remaining on nasal CPAP after the diagnosis. In our experience, all the neonates developed worsening respiratory severity in terms of increasing of respiratory rate, Silverman score, FiO_2_ requirement and PCO_2_ values, making intubation mandatory. HFOV was used as first choice of treatment (not as rescue, without a chest tube) and the strategy was found successful.

Our experience reported data on the successful use of HFOV to provide conservative management of a significant pneumothorax in preterm newborns. In our opinion this approach could be used in preterm newborns developing pneumothorax while they are on nasal CPAP, remaining hemodynamically stable without clinical signs of tension pneumothorax (i.e. cyanosis, significant decline of arterial blood pressure, heart rate, respiratory rate, and SpO_2_) [13] but requiring mechanical ventilation for acute worsening of the respiratory status. The suggested starting ventilator parameters in course of HFOV for pneumothorax treatment are: MAP 8–9 cm H_2_O, FiO_2_ to achieve SpO_2_ 90–95%, Respiratory Rate: 10 Hz, I:E = 1:2, ΔP: 15 cm H_2_O, eventually increased - chest to be «visibly vibrating ». This approach allowed us to avoid the increasing of air leak and the insertion of chest tube drainage and all the subsequent associated risks. However, there is no control population to which these 5 cases were compared, i.e. the pneumothorax may have resolved on their own regardless of ventilator modality. Nevertheless, considering both the reassuring outcomes observed in our babies and the risks of thoracic organ injury related to chest tube insertion because of supple chest wall, close proximity of vital structures and frail lung tissue of preterm babies, our experience could provide a starting point for novel hypothesis-testing clinical research (i.e. by comparing the conservative management of pneumothorax by HFOV or conventional mechanical ventilation in preterm infants).

Chest tube insertion for definitive drainage of a tension pneumothorax should be provided when this complication not only produces worsening of respiratory status (increase of respiratory rate, Silverman score, FiO_2_ requirement and PCO_2_ values), but also a hemodynamic instability of the patients (e.g. bradycardia, hypotension).

## Abbreviations

CPAP: Continuous positive airways pressure
HFOV: High-frequency oscillatory ventilation
HFV: High frequency ventilation
MAP: Mean airways pressure
